# Supplementary material for: LIT01-196, a Metabolically Stable Apelin-17 Analog, Normalizes Blood Pressure in Hypertensive DOCA-Salt Rats via a NO Synthase-dependent Mechanism
Source: Front Pharmacol. 2021 Jul 26;12:715095. doi: 10.3389/fphar.2021.715095 (PMC8359812; doi:10.3389/fphar.2021.715095)
Supplement: Supplementary file 1 [file Image2.pdf]

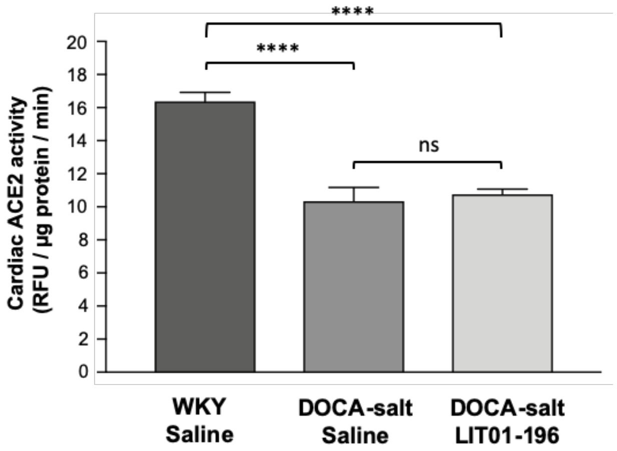

Supplementary Figure 2: Effects of the s.c. administration of LIT01-196 on cardiac ACE2 activity in hypertensive DOCA-salt rats.

Cardiac ACE2 activity was measured in normotensive WKY rats and in DOCA-salt hypertensive rats receiving saline or LIT01-196 (90 nmoles/kg) by s.c. route during 4 days (n=5 for each group). Cardiac ACE2 activity in DOCA-salt rats receiving LIT01-196 were compared with those of DOCA-salt rats receiving saline. The values in DOCA-salt rats were compared with those in normotensive WKY rats receiving s.c. injections of saline. The mean ± SEM values are presented for each set of conditions. One-way ANOVA followed by Sidak’s multiple comparisons test ****$P<0.0001$.
