# Supplementary material for: LIT01-196, a Metabolically Stable Apelin-17 Analog, Normalizes Blood Pressure in Hypertensive DOCA-Salt Rats via a NO Synthase-dependent Mechanism
Source: Front Pharmacol. 2021 Jul 26;12:715095. doi: 10.3389/fphar.2021.715095 (PMC8359812; doi:10.3389/fphar.2021.715095)
Supplement: Supplementary file 2 [file Image1.pdf]

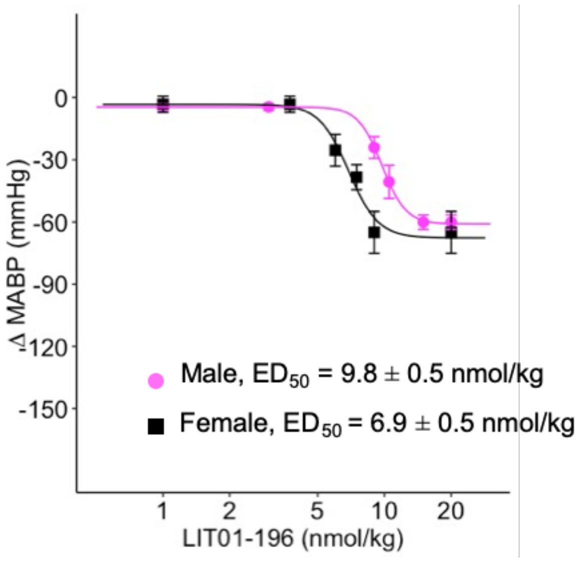


**Supplementary Figure 1: Effects of LIT01-196 given by intravenous route on arterial blood pressure in male and female Sprague-Dawley rats.**

Dose-response curve of LIT01-196 administered by the i.v. route, for MAPB changes in conscious SD normotensive male (n=18) and female (n=17) rats.
